# Francis John Crane Parsons

**Published:** 1906-03

**Authors:** 


### Obituary Notices.

### FRANCIS JOHN CRANE PARSONS, M.R.C.S., L.R.C.P.

This well-known and public-spirited townsman of Bridgwater died on the 10th of February, at the age of 58. He had been ill for some months, but he declined to give in to ordinary human ailments, and continued his work steadily till within three days of his death.

Frank Parsons, as he was always familiarly called, was the second son of a Bridgwater surgeon of considerable eminence, who had a large operative and consulting practice, and who was a man of strong character and marked ability.

Frank was educated at the Crewkerne Grammar School under Dr. Penny. He was a good cricketer, and was popular and generous. In those days our time in school was almost entirely given up to a study of Latin and Greek, and Parsons's verses and translations were, if not excellent, of the usual merit. His elder brother was a scholar at Eton, and passed high into the Indian Civil Service.

At about seventeen years of age Parsons entered King's College Hospital. He was a steady worker without being exceptionally clever or persevering. He was possessed of good common sense and of the power of observation, and although only two men in each year could be appointed to the post of House Physician, and Parsons was not working for University distinction, was in his year one of the two selected. The House Physician had at that time charge of all the medical beds in the hospital, the physicians being George Johnson, Baring Garrod, and Lionel Beale.

After such an experience, and with the introduction of his father, young Parsons rapidly gained a very extensive practice at and around Bridgwater. Possessed of good private means, there was no need for him to be overanxious, but he was fond of his profession and understood it, so that for many years he was much consulted, and until the time of his death he continued in active practice.

Frank Parsons had broad sympathies, and took an active interest in almost every matter that concerned the town of Bridgwater, of which he may well be said to have been a distinguished citizen, and there, as also among all his old fellow-students and colleagues, his memory will long be cherished.

